# Big data analytics in Cloud computing: an overview

**DOI:** 10.1186/s13677-022-00301-w

**Published:** 2022-08-06

**Authors:** Blend Berisha, Endrit Mëziu, Isak Shabani

**Affiliations:** Faculty of Electrical and Computer Engineering, Department of Computer Engineering, University of Prishtina, 10000 Prishtina, Kosovo

**Keywords:** Big data, Analytics, BigQuery, Cloud computing

## Abstract

Big Data and Cloud Computing as two mainstream technologies, are at the center of concern in the IT field. Every day a huge amount of data is produced from different sources. This data is so big in size that traditional processing tools are unable to deal with them. Besides being big, this data moves fast and has a lot of variety. Big Data is a concept that deals with storing, processing and analyzing large amounts of data. Cloud computing on the other hand is about offering the infrastructure to enable such processes in a cost-effective and efficient manner. Many sectors, including among others businesses (small or large), healthcare, education, etc. are trying to leverage the power of Big Data. In healthcare, for example, Big Data is being used to reduce costs of treatment, predict outbreaks of pandemics, prevent diseases etc. This paper, presents an overview of Big Data Analytics as a crucial process in many fields and sectors. We start by a brief introduction to the concept of Big Data, the amount of data that is generated on a daily bases, features and characteristics of Big Data. We then delve into Big Data Analytics were we discuss issues such as analytics cycle, analytics benefits and the movement from ETL to ELT paradigm as a result of Big Data analytics in Cloud. As a case study we analyze Google’s BigQuery which is a fully-managed, serverless data warehouse that enables scalable analysis over petabytes of data. As a Platform as a Service (PaaS) supports querying using ANSI SQL. We use the tool to perform different experiments such as average read, average compute, average write, on different sizes of datasets.

## Introduction

We live in the data age. We see them everywhere and this is due to the great technological developments that have taken place in recent years. The rate of digitalization has increased significantly and now we are rightly talking about” digital information societies”. If 20 or 30 years ago only 1% of the information produced was digital, now over 94% of this information is digital and it comes from various sources such as our mobile phones, servers, sensor devices on the Internet of Things, social networks, etc. [1]. The year 2002 is considered the” beginning of the digital age” where an explosion of digitally produced equipment and information was seen.

**Fig. 1:**
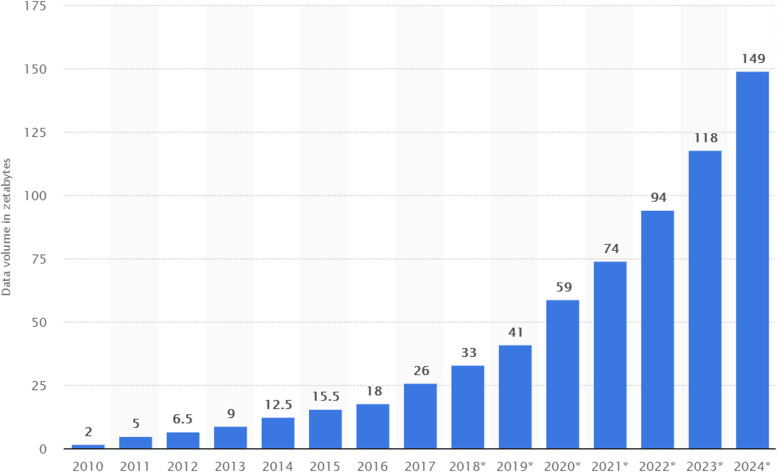
Volume of data/information created, captured, copied, and consumed worldwide from 2010 to 2024 (estimated) [3]

The number and amount of information collected has increased significantly due to the increase of devices that collect this information such as mobile devices, cheap and numerous sensor devices on the Internet of Things (IoT), remote sensing, software logs, cameras, microphones, RFID readers, wireless sensor networks, etc. [2]. According to statistics, the amount of data generated / day is about 44 zettabytes (44 × 10^21^ bytes). Every second, 1.7 MB of data is generated per person [3]. Based on International Data Group forecasts, the global amount of data will increase exponentially from 2020 to 2025, with a move from 44 to 163 zettabytes [4]. Figure 1 shows the amount of global data generated, copied and consumed. As can be seen, in the years 2010–2015, the rate of increase from year to year has been smaller, while since 2018, this rate has increased significantly thus making the trend exponential in nature [3].

To get a glimpse of the amount of data that is generated on a daily basis, let’s see a portion of data that different platforms produce. On the Internet, there is so much information at our fingertips. We add to the stockpile everytime we look for answers from our search engines. As a results Google now produces more than 500,000 searches every second (approximately 3.5 billion search per day) [5]. By the time of writing this article, this number must have changed! Social media on the other hand is a massive data producer. 

People’s ‘love affair’ with social media certainly fuels data creation. Every minute, Snapchat users share 527,760 photos, more than 120 professionals join LinkedIn, users watch 4,146,6000 Youtube videos, 456,000 are sent to Twitter and Instagram users post 46,740 photos [5]. Facebook remains the largest social media platform, with over 300 million photos uploaded every day with more than 510,000 comments posted and 293,000 statuses updated every minute.

With the increase in the number and quantity of data, there have been advantages but also challenges as systems for managing relational databases and other traditional systems have difficulties in processing and analyzing this quantity. For this reason, the term ‘big data’ arose not only to describe the amount of data but also the need for new technologies and ways of processing and analyzing this data. Cloud Computing has facilitated data storage, processing and analysis. Using Cloud we have access to almost limitless storage and computer power offered by different vendors. Cloud delivery models such as: IAAS (Infrastructure as a Service), PAAS (Platform as a Service) can help organisations across different sectors handle Big Data easier and faster. The aim of this paper is to provide an overview of how analytics of Big Data in Cloud Computing can be done. For this we use Google’s platform BigQuery which is a serverless data warehouse with built-in machine learning capabilities. It’s very robust and has plenty of features to help with the analytics of different size and type of data.

## What is big data?

Many authors and organizations have tried to provide a definition of ‘Big Data’. According to [6] “Big Data refers to data volumes in the range of exabytes and beyond”. In Wikipedia [7] big data is defined as an accumulation of datasets so huge and complex that it becomes hard to process using database management tools or traditional data processing applications, while the challenges include capture, storage, search, sharing, transfer, analysis, and visualization.

Sam Madden from Massachusetts Institute of Technology (MIT) considers” Big Data” to be data that is too big, too fast, or too hard for existing tools to process [8]. By too big, it means data that is at the petabyte level and that comes from various sources. By ‘too fast’ it means data growth which is fast and should also be processed quickly. By too hard it means the difficulty that arises as a result the data not adapting to the existing processing tools [9]. In PCMag (one of the most popular journals on technological trends), Big data refers to the massive amounts of data that is collected over time that are difficult to analyze and handle using common database management tools [10]. There are many other definitions for Big Data, but we consider that these are enough to gain an impression on this concept.

## Features and characteristics of big data

**Fig. 2:**
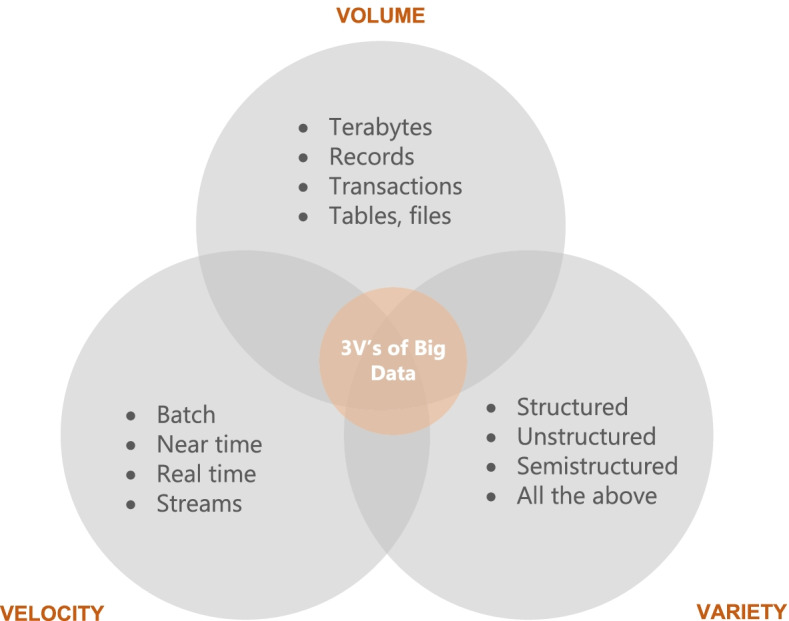
3 V’s of Big Data [6]

One question that researchers have struggled to answer is what might qualify as ‘big data’? For this reason, in 2001 industry analyst Doug Laney from Gartner introduced the 3 V model which are three features that must complement the data to be considered” big data”: *volume, velocity, variety*. *Volume* is a property or characteristic that determines the size of data, usually reported in Terabyte or Petabyte. For example, social networks like Facebook store among others photos of users. Due to the large number of users, it is estimated that Facebook stores about 250 billion photos and over 2.5 trillion posts of its users. This is an extremely large amount of data that needs to be stored and processed. Volume is the most representative feature of ‘big data’ [8]. In terms of volume, tera or peta level data is usually considered ‘big’ although this depends on the capacity of those analyzing this data and the tools available to them [8]. Figure 2 shows what each of the three V's represent.

**Fig. 3:**
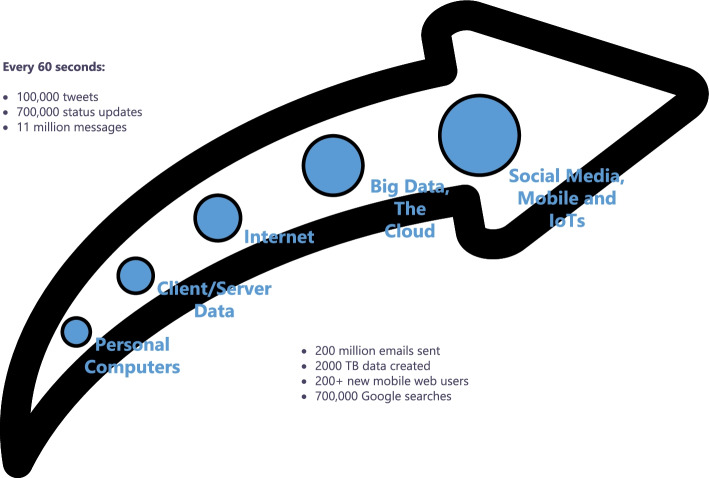
Examples of the velocity of Big Data [9]

The second property or characteristic is *velocity*. This refers to the degree to which data is generated or the speed at which this data must be processed and analyzed [8]. For example, Facebook users upload more than 900 million photos a day, which is approximately 104 uploaded photos per second. In this way, Facebook needs to process, store and retrieve this information to its users in real time. Figure 3 shows some statistics obtained from [11] which show the speed of data generation from different sources. As can be seen, social media and the Internet of Things (IoT) are the largest data generators, with a growing trend.

There are two main types of data processing: batch and stream. In batch, processing happens in blocks of data that have been stored over a period of time. Usually data processed in batch are big, so they will take longer to process. Hadoop MapReduce is considered to be the best framework for processing data in batches [11]. This approach works well in situations where there is no need for real-time analytics and where it is important to process large volumes of data to get more detailed insights.

Stream processing, on the other hand, is a key to the processing and analysis of data in real time. Stream processing allows for data processing as they arrive. This data is immediately fed into analytics tools so the results are generated instantly. There are many scenarios where such an approach can be useful such as fraud detection, where anomalies that signal fraud are detected in real time. Another use case would be online retailers, where real-time processing would enable them to compile large histories of costumer interactions so that additional purchases could be recommended for the costumers in real time [11].

**Fig. 4:**
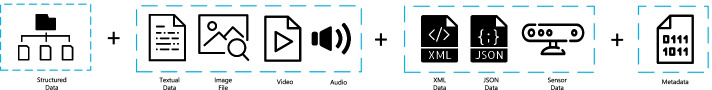
Main categories of data variety in Big Data [9]

The third property is *variety*, which refers to different types of data which are generated from different sources. “Big Data” is usually classified into three major categories: structured data (transactional data, spreadsheets, relational databases etc.), semi-structured (Extensible Markup Language - XML, web server logs etc) and unstructured (social media posts, audio, images, video etc.). In the literature, as a fourth category is also mentioned ‘meta-data’ which represents data about data. This is also shown in Fig. 4. Most of the data today belong to the category of unstructured data (80%) [11].

Over time, the tree features of big data have been complemented by two additional ones: *veracity* and *value*. Veracity is equivalent to quality, which means data that are clean and accurate and that have something to offer [12]. The concept is also related to the reliability of data that is extracted (e.g., costumer sentiments in social media are not highly reliable data). Value of the data is related to the social or economic value data can generate. The degree of value data can produce depends also on the knowledge of those that make use of it.

[12].

## Big data analytics in cloud computing

Cloud Computing is the delivery of computing services such as servers, storage, databases, networking, software, analytics etc., over the Internet (“the cloud”) with the aim of providing flexible resources, faster innovation and economies of scale [13]. Cloud computing has revolutionized the way computing infrastructure is abstracted and used. Cloud paradigms have been extended to include anything that can be considered as a service (hence *x* a service). The many benefits of cloud computing such as elasticity, pay-as-you-go or pay-per-use model, low upfront investment etc., have made it a viable and desirable choice for big data storage, management and analytics [13]. Because big data is now considered vital for many organizations and fields, service providers such as Amazon, Google and Microsoft are offering their own big data systems in a cost-efficient manner. These systems offer scalability for business of all sizes. This had led to the prominence of the term *Analytics as a Service (AaaS)* as a faster and efficient way to integrate, transform and visualize different types of data. Data Analytics.

### Big data analytics cycle

**Fig. 5:**
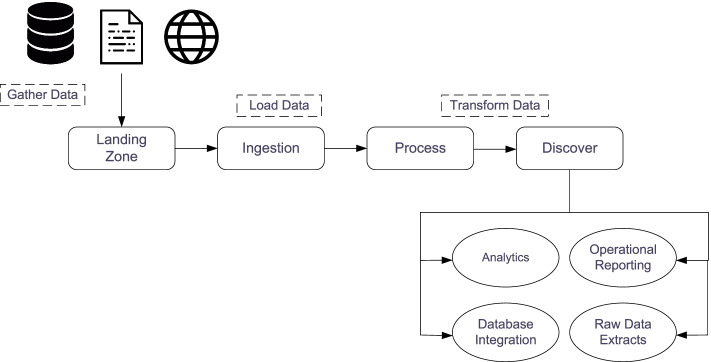
Flow in the processing of Big Data [11]

According to [14] processing big data for analytics differs from processing traditional transactional data. In traditional environments, data is first explored then a model design as well as a database structure is created. Figure 5. depicts the flow of big data analysis. As can be seen, it starts by gathering data from multiple sources, such as multiple files, systems, sensors and the Web. This data is then stored in the so called” landing zone” which is a medium capable of handling the volume, variety and velocity of data. This is usually a distributed file system. After data is stored, different transformations occur in this data to preserve its efficiency and scalability. Afer that, they are integrated into particular analytical tasks, operational reporting, databases or raw data extracts [14].

### Moving from ETL to ELT paradigm

ETL (Extract, Transform, Load) is about taking data from a data source, applying the transformations that might be required and then load it into a data warehouse to run reports and queries against them. The downside of this approach or paradigm is that is characterized by a lot of I/O activity, a lot of string processing, variable transformation and a lot of data parsing [15].

**Fig. 6:**
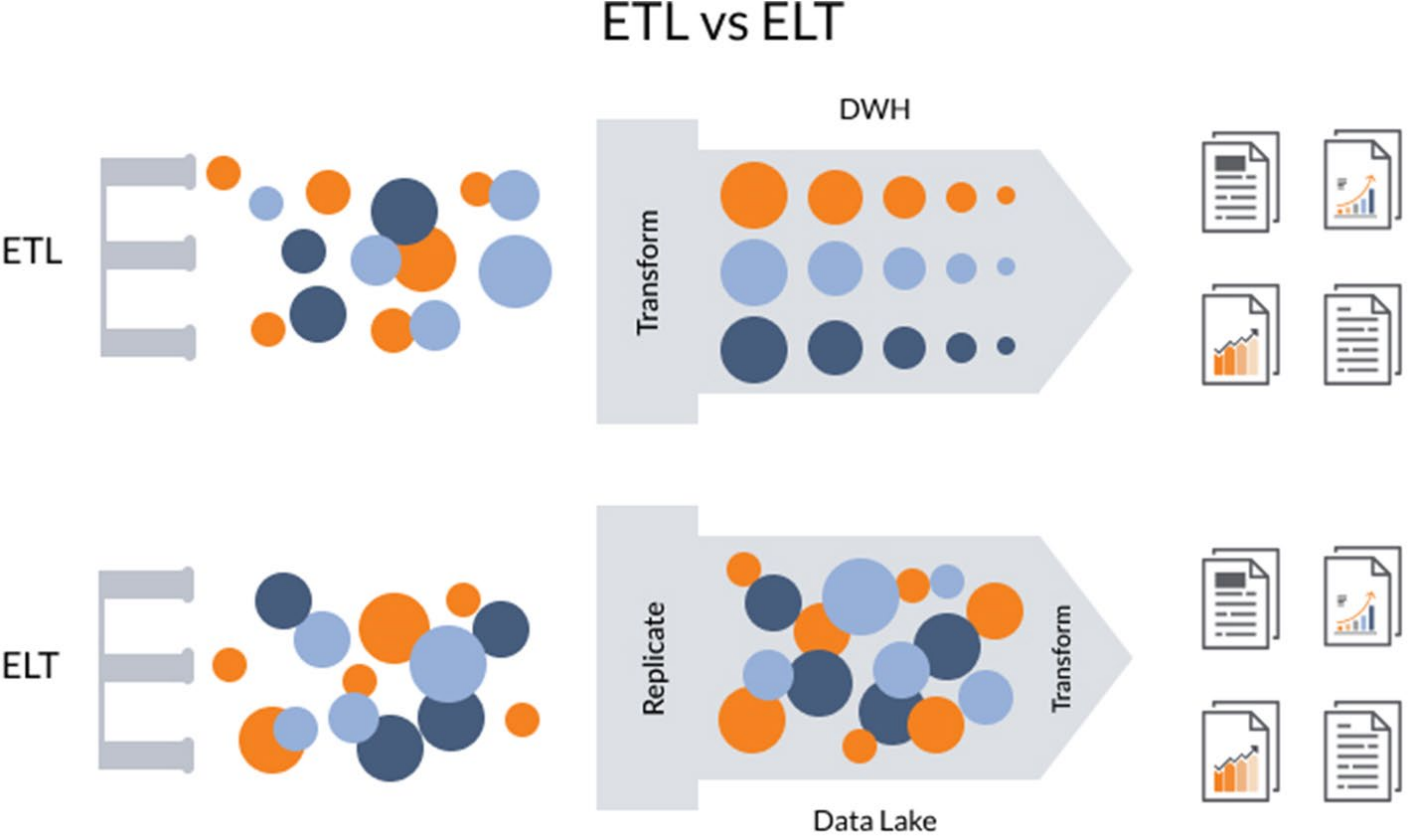
Differences between ETL and ELT [15]

ELT (Extract, Load, Transform) is about taking the most compute-intensive activity (transformation) and doing it not in an on-premise service which is already under pressure with regular transaction-handling but instead taking it to the cloud [15]. This means that there is no need for data staging because data warehousing solution is used for different types.

of data including those that are structured, semi-structured, unstructured and raw. This approach employs the concept of” data lakes” that are different from OLAP (Online Analytical Processing) data warehouses because they do not require the transformation of data before loading them [15]. Figure 6 illustrates the differences between the two paradigms. As seen, the main difference is where transformation process takes place.

ELT has many benefits over traditional ETL paradigm. The most crucial, as mentioned, is the fact that data of any format can be ingested as soon as it becomes available. Another one is the fact that only the data required for particular analysis can be transformed. In ETL, the entire pipeline and structure of the data in the OLAP may require modification if the previous structure does not allow for new types of analysis [16].

### Some advantages of big data analytics

As mentioned, companies across various sectors in the industry are leveraging Big Data in order to promote decision making that is data-driven. Besides tech industry, the usage and popularity of Big Data has expanded to include healthcare, governance, retail, supply chain management, education etc. Some of the benefits of Big Data Analytics mentioned in [17] include:

- Data accumulation from different sources including the Internet, online shopping sites, social media, databases, external third-party sources etc.
- Identification of crucial points that are hidden within large datasets in order to influence business decisions.
- Identification of the issues regarding systems and business processes in real time.
- Facilitation of service/product delivery to meet or exceed client expecations.
- Responding to customer requests, queries and grievances in real time.

Some other benefits according to [16] are related to:

1. *Cost optimization -* One of the biggest advantages of Big Data tools such as Hadoop or Spark is that they offer cost advantages to businesses regarding the storage, processing and analysis of large amounts of data. Authors mention the logistics industry as an example to highlight the cost-reduction benefits of Big Data. In this industry, the cost of product returns is 1.5 times higher than that of actual shipping costs. With Big Data Analytics, companies can minimize product return costs by predicting the likelihood of product returns. By doing so, they can then estimate which products are most likely to be returned and thus enable the companies to take suitable measures to reduce losses on returns.
2. *Efficiency improvements -* Big Data can improve operational efficiency by a margin. Big Data tools can amass large amounts of useful costumer data by interacting and gaining their feedback. This data can then be analyzed and interpreted to extract some meaningful patterns hidden within such as customer taste and preferences, buying behaviors etc. This in turn allows companies to create personalized or tailored products/services.
3. *Innovation -* Insights from Big Data can be used to tweak business strategies, develop new products/services, optimize service delivery, improve productivity etc. These can all lead to more innovation.

As seen, Big Data Analytics has been mostly leveraged by businesses, but other sectors have also benefited. For example, in healthcare many states are now utilizing the power of Big Data to predict and also prevent epidemics, cure diseases, cut down costs etc. This data has also been used to establish many efficient treatment models. With Big Data more comprehensive reports were generated and these were then converted into relevant critical insights to provide better care [17].

In education, Big Data has also been used extensively. They have enabled teachers to measure, monitor and respond in real-time to student’s understanding of the material. Professors have created tailor-made materials for students with different knowledge levels to increase their interest [18].

## Case study: GOOGLE’S big query for data processing and analytics

Google Cloud Platform contains a number of services designed to analyze and process big data. Throughout this paper we have described and discussed the architecture and main components of Biguery as one of the most used big data processing tools in GCP. BigQuery is a fully-managed, serverless data warehouse that enables scalable analysis over petabytes of data. It is a Platform as a Service (PaaS) that supports querying using ANSI SQL. It also has built-in machine learning capabilities. Since its launch in 2011 it has gained a lot of popularity and many big companies have utilized it for their data analytics [19].

**Fig. 7:**
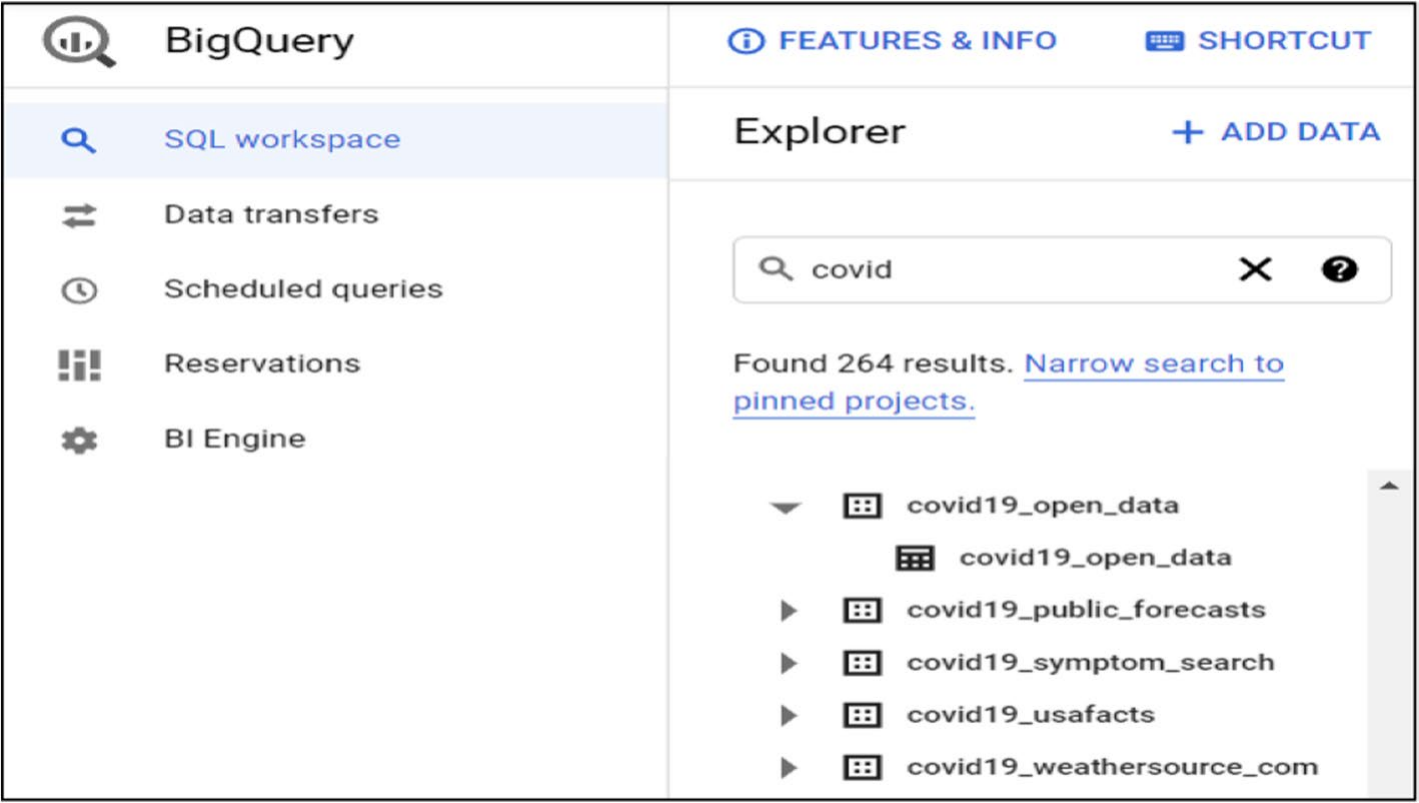
BigQuery Interface

From a user perspective, BigQuery has an intuitive user interface which can be accessed in a number of ways depending on user needs. The simplest way to interact with this tool is to use its graphical web interface as shown in Fig. 7. Slightly more complicated but faster approaches include using cloud console or Bigquery APIs. From Fig. 7 Bigquery web interface offers you the options to add or select existing datasets, schedule and construct queries or transfer data and display results.

**Fig. 8:**
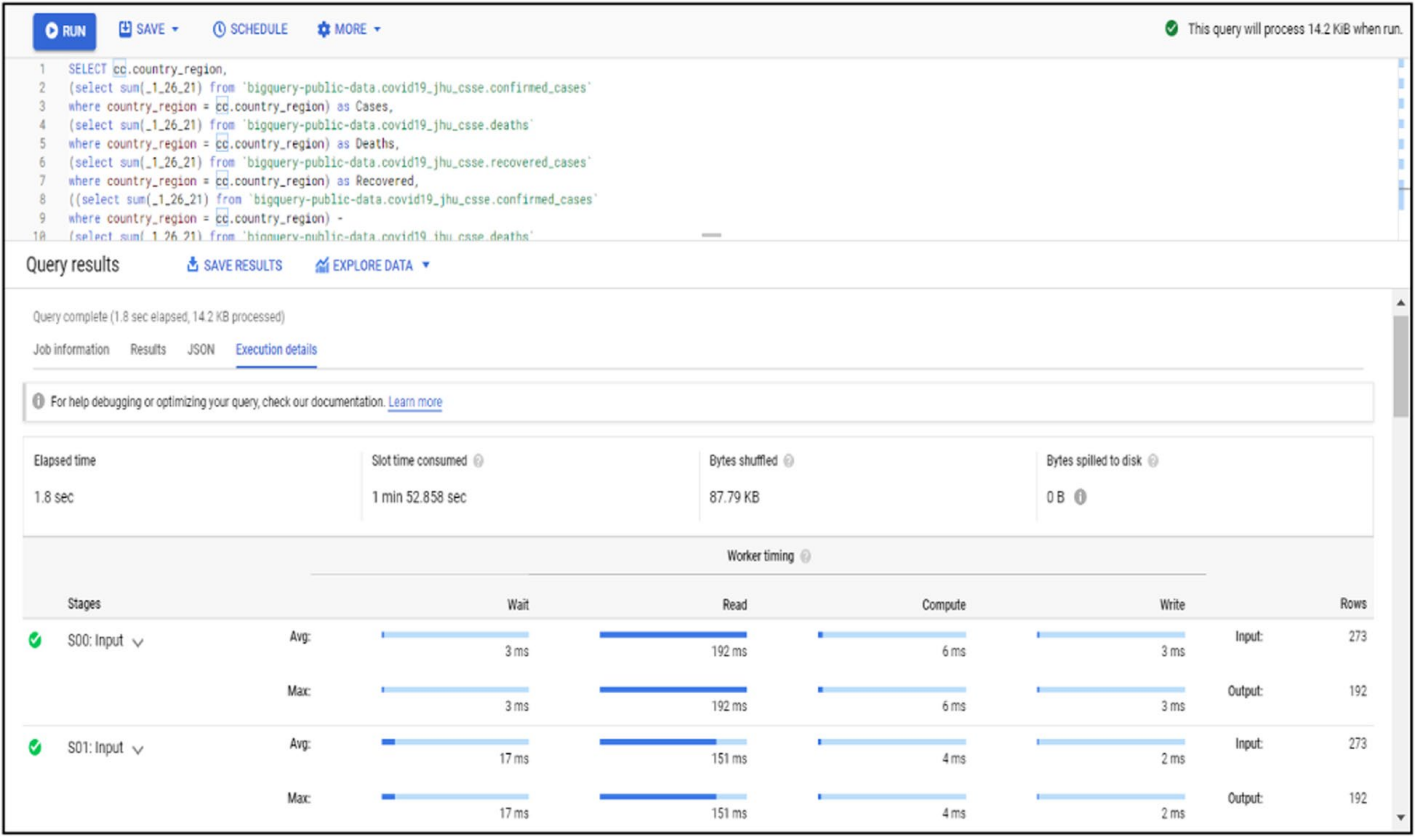
BigQuery execution details

**Fig. 9:**
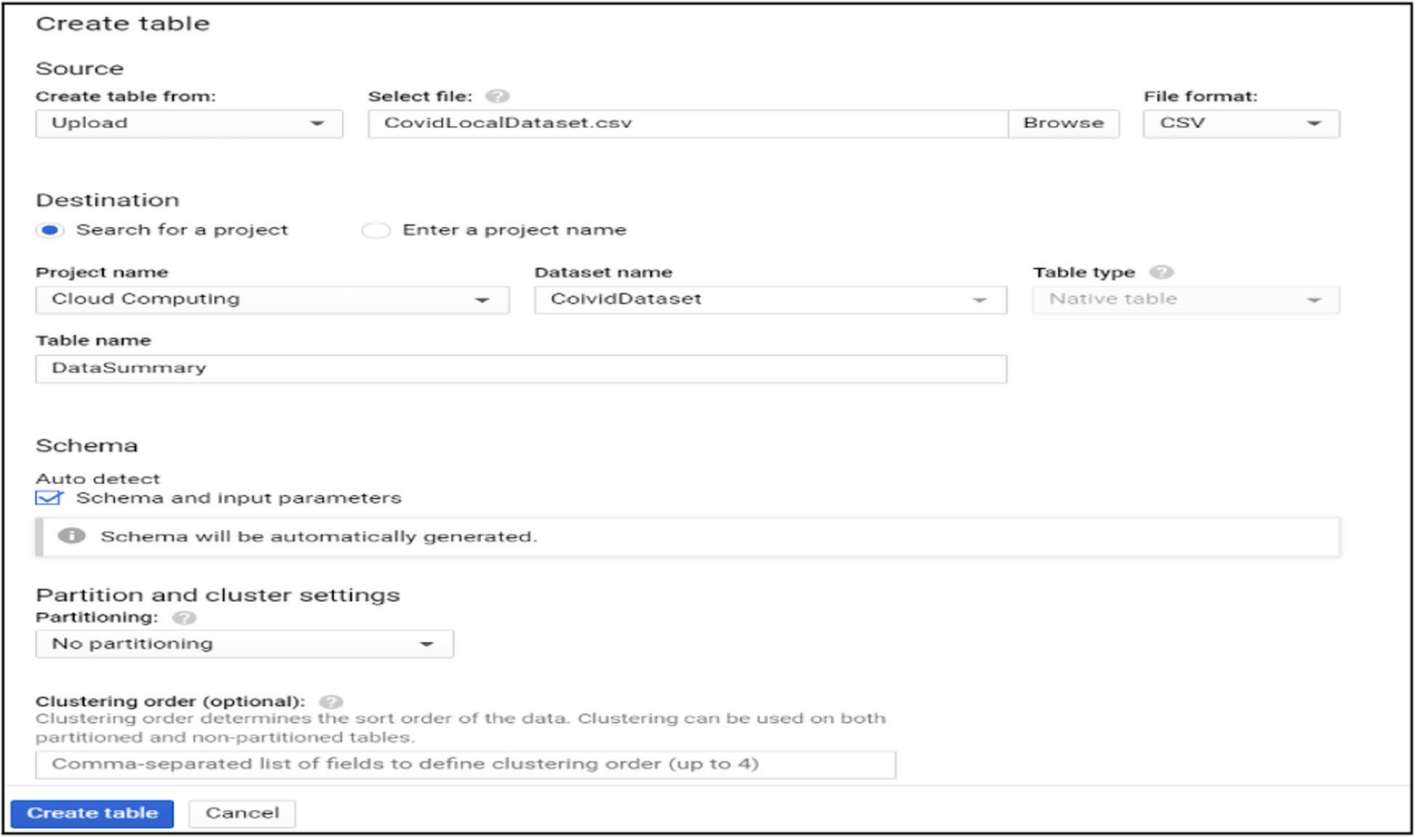
Adding table to the created dataset

Data processing and query construction occurs under the sql workspace section, Bigquery offers a rich sql-like syntax to compute and process large sets of data, it operates on relational datasets with well-defined structure including tables with specified columns and types. Figure 8 shows a simple query construction syntax and highlights its execution details. Data displayed under query results shows main performance components of the executed query starting from elapsed time, consumed slot time, size of data processed, average and maximum wait, write and compute times. Query defined in Fig. 8 combines three datasets which contain information regarding Covid-19 reported cases, deaths and recoveries from more than 190 countries through year 2020 till January 2021. Google BigQuery is flexible in a way that allows you to use and combine various datasets suitable for your task easily and with small delays. It contains an ever growing list of public datasets at your disposal and also offers the options to create, edit and import your own. Figure 9 shows the process of adding a table to the newly created dataset. From the Fig. 9, we see that for table creation as a source we have used a local csv file, this file will be used to create table schema and populate it with data, aside from local upload option as a source to create the table we can use Google BigTable, Google Cloud Storage or Google Drive. The newly created table with its respective data then is ready to be used to construct queries and obtain new insights as shown in Fig. 8.

**Fig. 10:**
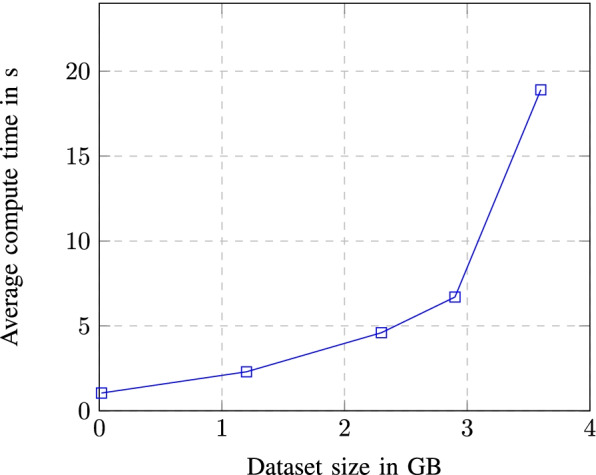
Average compute time dependence in dataset size

One advantage of using imported data in the cloud is the option to manage its access and visibility in the cloud project and cloud members scope. Depending from the way of use, queried data can be saved directly to the local computer through the use of “save results” option from Fig. 8 which offers a variety of formats and data extensions settings to choose from but can also be explored in different configurations using “explore data” option. You can also save constructed queries for later use or schedule query execution interval for more accurate data transmutation through API endpoints. Figure 10 shows how much the average compute time will change/increase with the increase in the size of the dataset used.

### Experiments with different dataset sizes

Before moving to data exploration lets analyze performance results of BigQuery in simple queries with variable dataset sizes. In Table 1 we have shown the query execution details of five simple select queries done on five different datasets. The results are displayed against six different performance categories, from the data we see a correlation between size of the dataset and its average read, write and compute.

**Table 1:** BigQuery performance tests

Elapsed Time (s)	Slot Time Consumed (s)	Average Read (ms)	Average Compute (s)	Average Write (ms)	Number Of Rows	Size Of The Dataset
0.3	0.043	22	1.048	2	61,900	0.0175 GB
72	828.547	355	2.3	28,599	41,340	1.2 GB
3.3	3.663	945	4.6	109	100,000	2.3 GB
2.1	2.424	118	6.7	77	30,646	2.9 GB
1.6	1.506	237	18.9	145	100,000	3.6 GB

From the graph we see that the dependence between dataset size and average compute size is exponential, meaning that with the increase in data size, average compute time is exponentially increased.

**Fig. 11:**
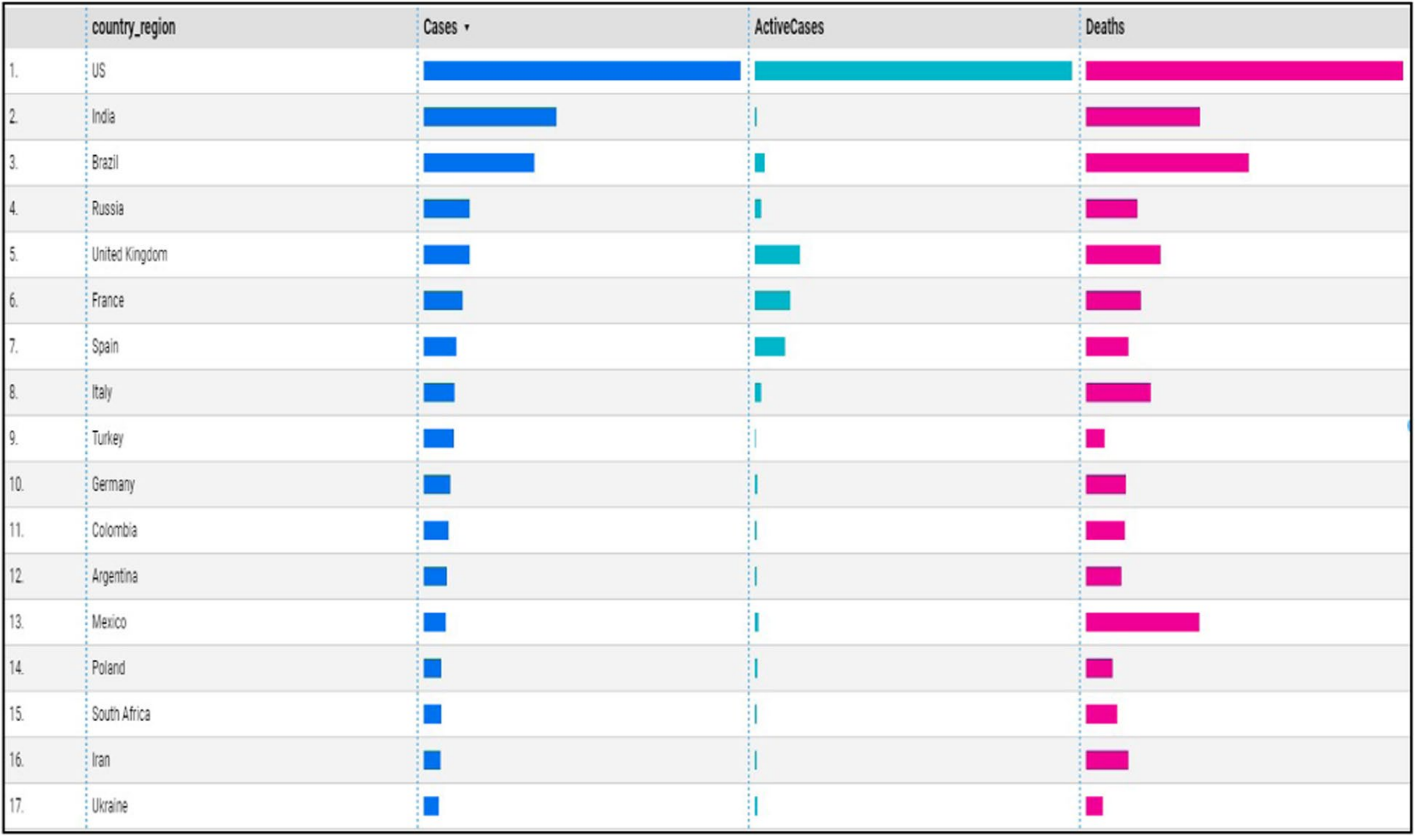
Using data studio for data visualization

Data returned from constructed queries aside from being displayed in a simple tabular form or as a JSON object can also be transferred to data studio which is an integrated tool to better display and visualize gathered information. One way of displaying queried data from Fig. 8 with data studio tool is shown in Fig. 11. In this case a bar table chart visualization option is chosen.

## Conclusion

Big Data is not a new term but has gained its spotlight due to the huge amounts of data that are produced daily from different sources. From our analysis we saw that big data is increasing in a fast pace, leading to benefits but also challenges. Cloud Computing is considered to be the best solution for storing, processing and analyzing Big Data. Companies like Amazon, Google and Microsoft offer their public services to facilitate the process of dealing with Big Data. From the analysis we saw that there are multiple benefits that Big Data analytics provides for many different fields and sectors such as healthcare, education and business. We also saw that because of the interaction of Big Data with Cloud Computing there is a shift in the way data is processed and analyzed. In traditional settings, ETL is used whereas in Big Data, ELT is used. We saw that the latter has clear advantages when compared to the former.

From our case study we saw that BigQuery is very good for running complex analytical queries, which means there is no point in running queries that are doing simple aggregation or filtering. BigQuery is suitable for heavy queries, those that operate using a big set of data. The bigger the dataset, the more it is likely to gain in performance. This is when compared to the traditional relational databases,as BigQuery implements different parallel schemas to speed up the execution time.

BigQuery doesn’t like joins and merging data into one table gets a better execution time. It is good for scenarios where data does not change often as it has built-in cache. BigQuery can also be used when one wants to reduce the load on the relational database as it offers different options and configurations to improve query performance. Also pay as you go service can be used where charges are made based on usage or flat rate service which offers a specific slot rate and charges in daily, monthly or yearly plan.

## Data Availability

The datasets used during the current study are available from the corresponding author on reasonable request. The authors declare that they have no funder.

## References

[1] Hillbert M, Lopez P (2011). The world’s technological capacity to store, communicate and compute information. Science.

[2] Hellerstein J (2019). Gigaom Blog.

[3] Statista (2020). Statista.

[4] Reinsel D, Gantz J, Rydning J (2017). Data age 2025: the evolution of data to-life critical.

[5] Forbes (2020). Forbes.

[6] Kaisler S, Armour F, Espinosa J (2013) Big data: issues and challenges moving forward, Wailea, Maui, HI, s.n, pp 995–1004

[7] Wikipedia (2018). Wikipedia.

[8] Gewirtz D (2018). ZDNet.

[9] Weathington J (2012) Big Data Defined. Tech Republic. https://www.techrepublic.com/article/big-data-defined/

[10] PCMagazine,“ PC Magazine,” 2018. Available: http://www.pcmag.com/encyclopedia/term/62849/big-data. Accessed 9 Jan 2021

[11] Akhtar SMF (2018). Big Data Architect’s Handbook, Packt.

[12] WhishWorks (2019). WhishWorks.

[13] Yadav S, Sohal A (2017) Review paper on big data analytics in Cloud computing. Int J Comp Trends Technol (IJCTT) IX. 49(3);156-160

[14] Kimball R, Ross M (2013). The data warehouse toolkit: the definitive guide to dimensional modeling.

[15] LaprinthX (2018). LaprinthX.

[16] Xplenty (2019). XPlenty.

[17] Forbes (2018). Forbes.

[18] EDHEC (2019). EDHEC.

[19] Google Cloud (2020). BigQuery.

